# Case report: mismatch repair proficiency and microsatellite stability in gastric cancer may not predict programmed death-1 blockade resistance

**DOI:** 10.1186/s13045-016-0259-0

**Published:** 2016-03-24

**Authors:** Kuo-Hsing Chen, Chang-Tsu Yuan, Li-Hui Tseng, Chia-Tung Shun, Kun-Huei Yeh

**Affiliations:** Department of Oncology, National Taiwan University Hospital, 7, Chun-Shan S Rd, Taipei, 10002 Taiwan; Department of Pathology, National Taiwan University Hospital, Taipei, Taiwan; Department of Medical Genetics, National Taiwan University Hospital, Taipei, Taiwan; National Taiwan University Cancer Center, Taipei, Taiwan; Department of Forensic Medicine, National Taiwan University, Taipei, Taiwan; Graduate Institute of Oncology, National Taiwan University, Taipei, Taiwan; Forensic Medicine, College of Medicine, National Taiwan University, Taipei, Taiwan

**Keywords:** Gastric cancer, Immunotherapy, Anti-programmed death-1 antibody, Mismatch repair deficiency, Mismatch repair proficiency, Microsatellite stability

## Abstract

**Background:**

Anti-programmed death-1 therapy has poor efficacy in mismatch repair-proficient (pMMR) colorectal cancers; however, its efficacy in pMMR gastric cancers remains undetermined. Here, we report the case of a patient with pMMR and microsatellite-stable gastric cancer who exhibited a partial response to salvage anti-programmed death-1 therapy with pembrolizumab.

**Case presentation:**

Initially, the patient underwent subtotal gastrectomy 4 years ago for early-stage gastric cancer (pT1bN2M0, stage IIA). Immunohistochemical analysis of the tumor revealed strongly positive for HER2/neu. He had received trastuzumab plus pertuzumab, cisplatin, and capecitabine for recurrent tumors since September 2014 for 15 cycles. Disease progression of gastric cancer was found in August 2015. Since September 2015, the patient has received pembrolizumab monotherapy (200 mg as a fixed dose, every 3 weeks) for 3 months and the repeat computed tomography demonstrated a confirmed partial response. The plasma carcinoembryonic antigen also decreased dramatically. Both immunohistochemistry and a polymerase chain reaction-based method revealed that the patient had pMMR gastric cancer.

**Conclusions:**

This case report provides the first report that mismatch repair-proficient and microsatellite-stable gastric cancers can respond well to anti-PD-1 monotherapy and indicates both markers may not sufficiently be predictive of anti-PD-1 therapy resistance in gastric cancer.

## Background

Immune checkpoint blockade therapies such as those using anti-cytotoxic T lymphocyte-associated protein 4 antibodies and those blocking programmed death-1 (PD-1) and programmed death ligand-1 (PD-L1) have exhibited promising efficacy in several types of cancer [1–7]. Moreover, the heterogeneous response to immune checkpoint blockade therapy observed in patients in these studies has raised the importance of identifying predictive biomarkers. Mutation or hypermethylation in mismatch repair genes (*MLH1*, *MSH2*, *MSH6*, or *PMS2*) leads to deficient mismatch repair (dMMR) and causes accumulation errors in DNA sequences, thus increasing the risk of colorectal cancer (CRC) and other epithelial cancers [8]. Recently, Le et al. reported that patients with mismatch repair-deficient CRC and those with mismatch repair-deficient noncolorectal cancer exhibited higher response rates to PD-1 blockade monotherapy (pembrolizumab). In addition, they suggested that hypermutated tumor-associated neoantigens from dMMR are the primary factors affecting the response to anti-PD-1 therapy; previous studies have observed that this therapy stimulated the endogenous immune response [3, 9, 10]. However, Le et al. also reported that none of 18 patients with mismatch repair-proficient (pMMR) CRC responded to pembrolizumab, an anti-PD-1 antibody. The efficacy of PD-1 blockade therapy in pMMR noncolorectal cancers and a biomarker for PD-1 blockade remain unknown. Here, we report the case of a 64-year-old man with pMMR and microsatellite-stable (MSS) gastric cancer who exhibited a partial response to salvage pembrolizumab monotherapy.

## Case presentation

**Fig. 1:**
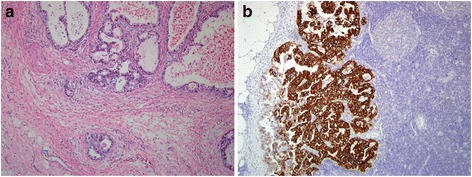
Histopathology of the gastric cancer and its HER2 immunohistochemistry. Microscopic observation revealed a well-differentiated adenocarcinoma with frequent luminal formation and evident nuclear atypia infiltration within a mild desmoplastic stroma; H & E stain (**a**). In the metastasized lymph node, the tumor cells were strongly positive for the HER2/neu immunostain (*brown color*) (**b**); original magnification ×100

A 64-year-old man experienced epigastralgia with tarry stool in late 2011. Esophagogastroduodenoscopy revealed a circular ulcer in the prepyloric area. Biopsy was performed, and the pathology of the specimen revealed gastric adenocarcinoma. On the basis of computed tomography (CT), the gastric cancer was clinically staged as cT3N0M0, stage IIA. He underwent radical subtotal gastrectomy, Billroth II anastomosis, D2 dissection, and cholecystectomy on December 28, 2011. The pathology of the specimen revealed a well-differentiated adenocarcinoma, and *Helicobacter pylori* infection was not observed (Fig. 1a). Immunohistochemically, tumor cells were strongly positive (3+, according to the proposal by Hofmann et al. [11]) for HER2/neu immunostain (clone 4B5, 1:2 dilution; Fig 1b). The pathological staging of the gastric cancer was pT1bN2M0, stage IIA. After surgery, he was followed up regularly at our hospital without adjuvant treatment.

Two years later, CT and positron emission tomography revealed tumor recurrence in the left lower paratracheal lymph nodes. The patient was then enrolled in a clinical trial and received 15 cycles of pertuzumab (840 mg on D1, every 3 weeks) in combination with trastuzumab (562 mg on D1, every 3 weeks) and chemotherapy (80 mg/m^2^ cisplatin on D1 and 1000 mg/m^2^ capecitabine twice a day on D1–D14, every 3 weeks). He was dropped from the study in August 2015 because the tumors (peritoneal seeding and paraaortic lymph nodes) progressed and his plasma carcinoembryonic antigen (CEA) level increased from 4.78 to 348 ng/mL. The best response of the treatment was stable disease with a first-line progression-free survival of 11 months.

**Fig. 2:**
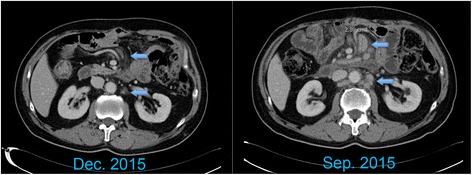
Computed tomography scan of the recurrent tumors before and after pembrolizumab treatment

**Fig. 3:**
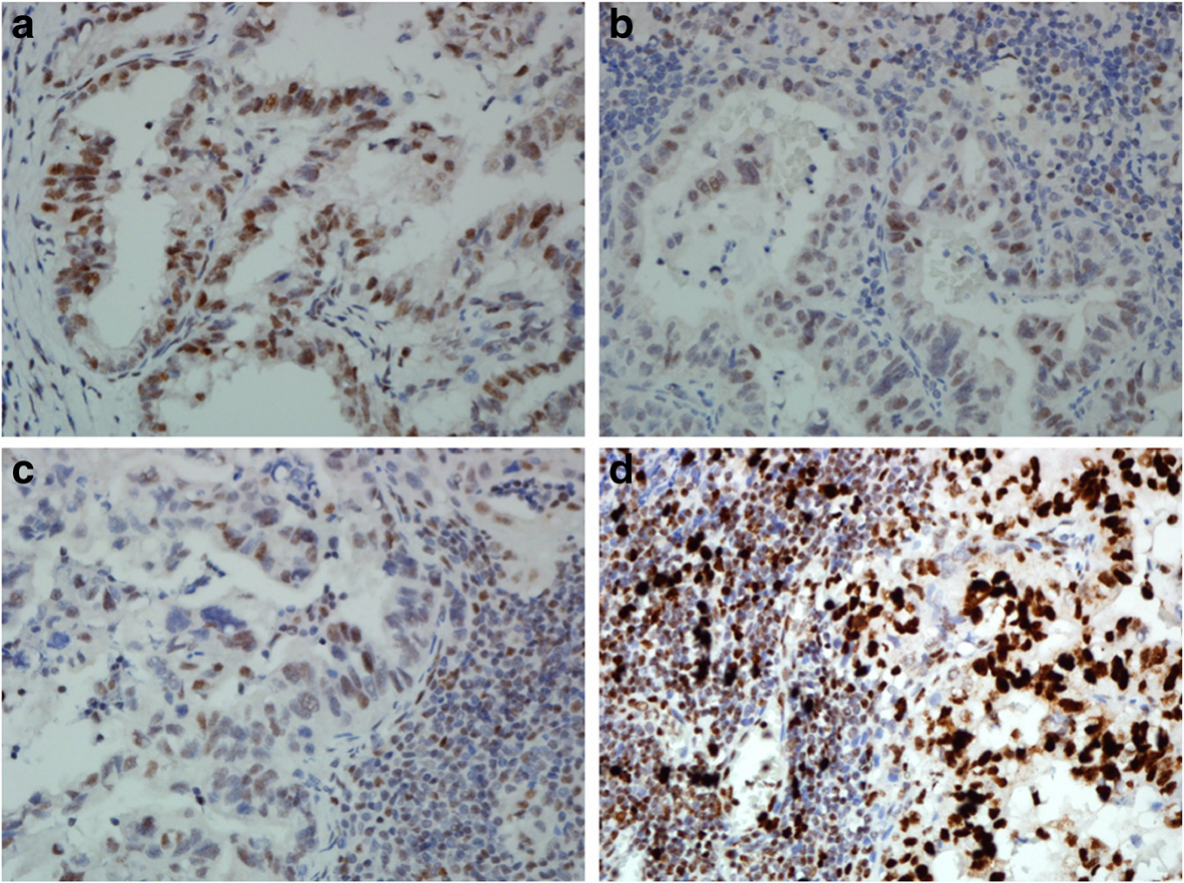
Immunohistochemistry of MMR protein. The tumor cells preserved expression of MLH1 (**a**), MSH2 (**b**), PMS2 (**c**), and MSH6 (**d**); original magnification ×400

**Fig. 4:**
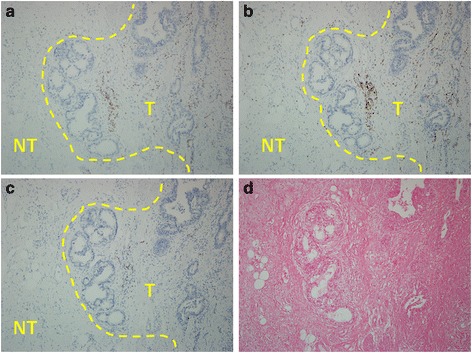
Tumor-infiltrating lymphocytes and EBV status. The invasive front is highlighted by a *yellow dashed line*, which separates the tumor part (*T*) from the nontumor part (*NT*). Few CD3(+) tumor-infiltrating lymphocytes (*brown spot*) (**a**). The infiltrating CD3(+) cells were composed mainly of CD4(+) cells (*brown spot*) (**b**) and a few CD8(+) cells (*brown spot*) (**c**). The invasive front had only a few lymphocytes. Most of the lymphocytes were scattered between tumor nests rather than within tumor nests. EBER in situ hybridization revealed no evidence of EBV infection in the tumor cells (positive is denoted by a dark blue spot in nuclei) (**d**); original magnification: ×100

In September 2015, the patient started receiving pembrolizumab (200 mg as a fixed dose, every 3 weeks) as a second-line treatment for recurrent gastric cancer. No adverse event was observed in the following 2–3 months. Two months later, CT revealed tumor regression and a partial response was confirmed in another CT scan 1 month later (Fig. 2). In addition, the plasma CEA level decreased from 607.1 to 26.96 ng/mL. To investigate the association of the tumor factors with the efficacy of PD-1 blockade as well as to confirm whether he had pMMR gastric cancer, we used both immunohistochemistry (IHC) and a polymerase chain reaction-based method (Fig. 3) [12]. In addition, we performed IHC analyses of previous specimens from gastrectomy, which revealed few tumor-infiltrating lymphocytes (TILs) in tumors and invasive fronts, as demonstrated by the presence of CD3 (polyclonal, 1:100), CD4 (clone MT310, 1:50), and CD8 (clone DK-25, 1:200; Fig. 4a–c). Epstein–Barr virus (EBV)-encoded small RNA (EBER) in situ hybridization revealed absence of EBV in the tumor cells (Fig. 4d). The tumor cells were also negative for PD-L1 (B7H1, Abcam, clone ab58810, 1:400; Fig. 5) [13]. Moreover, no polymerase epsilon (*POLE*) mutation was found by means of Sanger sequencing of exon 9. As of January 2016, the patient was still continuing the pembrolizumab monotherapy with excellent performance status and quality of life.

**Fig. 5:**
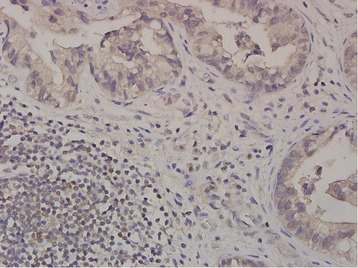
Immunohistochemistry of PD-L1 protein. The tumor cells were negative for PD-L1 (B7-H1); original magnification ×400

### Discussion

Evaluations of the efficacy of PD-1 blockade in advanced esophagogastric junction (EGJ) and gastric adenocarcinoma are still preliminary, and an accurate predictive biomarker remains to be determined. Bang et al. reported that the overall response rate of pembrolizumab (by central review) in advanced PD-L1-positive gastric cancer was 22.2 % [95 % confidence interval, 10.1–39.2] in a phase I study (KEYNOTE-012 study, gastric cancer cohort) [14]. Of the 162 patients screened, 65 were positive for PD-L1. The PD-L1 expression was detected through a prototype IHC assay by using the 223C antibody, and the tumors were determined positive for PD-L1 when PD-L1 staining was observed in the stroma or ≥1 % of tumor nest cells. However, PD-L1 expression is not yet a validated predictive biomarker for PD-1 blockade in gastric cancer. Moreover, dMMR has been identified in gastric cancer in several studies with the incidence ranging from 9 to 22 % [15–19]. In a study conducted using the Cancer Genome Atlas (TCGA), 22 % of the tumors belonged to the microsatellite instability (MSI) subtype, with the features of hypermutation and *MLH1* silencing. Although pMMR has been shown to predict poor efficacy of PD-1 blockade in CRC, the association of pMMR and PD-1 blockade in EGJ and gastric adenocarcinoma remains unknown. This case report provides the first report that pMMR and MSS gastric cancers can respond well to anti-PD-1 monotherapy. However, larger studies are warranted to explore the association between mismatch repair status and the efficacy of anti-PD-1 therapy in advanced gastric cancer.

The hypothesis that neoantigens from hypermutated tumors may enhance the response of immune checkpoint therapy was supported by clinical correlative studies on melanoma and non-small-cell lung cancer [3, 20, 21]. In addition to dMMR, inactivating *POLE* mutation, found in CRC, endometrial cancer, and gastric cancer, can result in an extremely high mutation burden [15, 22, 23]. Howitt et al. demonstrated that increased TILs in the tumor microenvironment in *POLE*-mutated endometrial cancers make these tumors satisfactory candidates for immune checkpoint therapy [23]. We cannot completely exclude the possibility that our patient had a *POLE*-mutated tumor; however, the incidence of *POLE* mutation in gastric cancer is very low (0.47 %) and TILs in the tumor microenvironment were scant in our patient [15].

PD-L1 has been reported to be overexpressed in EBV-associated malignancies, such as EBV-associated lymphoproliferative diseases, nasopharyngeal carcinoma, and HHV8-associated primary effusion lymphoma [24, 25]. The mechanisms underlying increased PD-L1 expression in Hodgkin lymphoma include genetic amplification of *CD274* (encoding PD-L1) and constitutive AP1 signaling. The TCGA study also revealed that the amplification of *CD274* and *PDCD1LG2* (also encoding PD-L1 and PD-L2) was enhanced in the EBV-positive gastric cancer subgroup [15]. Thus, these cancers are attractive targets for immune checkpoint therapy. In our patient, EBER in situ hybridization revealed absence of EBV in the tumor cells, and it is unlikely that the efficacy of anti-PD-1 therapy is affected by EBV-related *CD274* and *PDCD1LG2* amplification.

## Conclusions

In conclusion, we report the case of a patient with pMMR and MSS gastric cancer who exhibited a confirmed objective response to PD-1 blockade, pembrolizumab monotherapy. Our case report indicates that mismatch repair proficiency and microsatellite stability may not be predictive to resistance of anti-PD-1 therapy, and factors other than dMMR and EBV infection may contribute to the response to anti-PD-1 therapy.

### Consent for publication

Written informed consent was obtained from the patient for publication of this case report and any accompanying images. A copy of the written consent is available for review by the Editor-in-Chief of this journal. The institutional review board of National Taiwan University Hospital approved the study.

## Abbreviations

CRC: colorectal cancer
CT: computed tomography
dMMR: deficient mismatch repair
EBV: Epstein–Barr virus
EBER: Epstein–Barr virus-encoded small RNA
EGJ: esophagogastric junction
IHC: immunohistochemistry
PD-1: programmed death-1
PD-L1: programmed death ligand-1
pMMR: mismatch repair-proficient
MSI: microsatellite instability
MSS: microsatellite stable
POLE: polymerase epsilon
TCGA: the Cancer Genome Atlas
TILs: tumor-infiltrating lymphocytes
